# Derrone Inhibits Platelet Aggregation, Granule Secretion, Thromboxane A_2_ Generation, and Clot Retraction: An *In Vitro* Study

**DOI:** 10.1155/2021/8855980

**Published:** 2021-03-10

**Authors:** Jung-Hae Shin, Muhammad Irfan, Man Hee Rhee, Hyuk-Woo Kwon

**Affiliations:** ^1^Department of Biomedical Laboratory Science, Catholic Kwandong University, Gangneung 25601, Republic of Korea; ^2^Laboratory of Physiology and Cell Signaling, College of Veterinary Medicine, Kyungpook National University, Daegu 41566, Republic of Korea; ^3^Department of Oral Biology, University of Illinois at Chicago, Chicago 60607, IL, USA; ^4^Department of Biomedical Laboratory Science, Far East University, Eumseong 27601, Republic of Korea

## Abstract

*Cudrania tricuspidata* (*C. tricuspidata*) is widespread throughout East Asia and in China and Korea, and it is widely used as a traditional remedy against eczema, mumps, and tuberculosis. With regard to the aforementioned medical efficacy, various studies are continuously being conducted, and it has been reported that *C. tricuspidata* extract has various actions against inflammation, diabetes, obesity, and tumors. Therefore, we evaluated antiplatelet effects using derrone in *C. tricuspidata.* We examined the effect of derrone on the phosphorylation of vasodilator-stimulated phosphoprotein (VASP) and inositol 1, 4, 5-triphosphate receptor I (IP_3_RI), and on the dephosphorylation of cytosolic phospholipase A_2_ (cPLA_2_), mitogen-activated protein kinases p38 (p38^MAPK^), and Akt, which affects platelet function and thrombus formation. Various agonists-induced human platelets were inhibited by derrone without cytotoxicity, and it also decreased the intracellular calcium level through the signaling molecule phosphorylations. In addition, derrone inhibited glycoprotein IIb/IIIa (*α*IIb/*β*3) affinity. Thus, in the present study, derrone suppressed human platelet aggregation and thrombin-induced clot formation.

## 1. Introduction

In normal circulation of blood, platelets are necessary for hemostasis and thrombosis. [1]. The cardiovascular diseases (CVDs) are becoming a critical threat to our lives in these years. It is now widely accepted that platelets play an important role in cardiovascular disease as they have a fundamental role in thrombosis. Therefore, many drugs or natural substances have been developed to treat CVDs. [2, 3]. Inhibition of platelets has influence on the CVDs; however, the survival rate is still low. Thus, discovery of various substances is required [4]. In addition, there is an ever-increasing interest toward different traditional medicines (mainly, herbal remedies) in different diseases, including CVDs [5–7]. Various ginsenosides have been used as a traditional herbal medicine against several diseases, especially CVDs [8]. Reviewing these options could enable scientists to examine and then introduce novel efficacious medicines.

In normal circulation, synthesized cyclic AMP (cAMP) and cyclic GMP (cGMP) in platelets downregulate platelet function through cAMP/cGMP-dependent kinases, protein kinase A (PKA), and protein kinase G (PKG) [9]. Vasodilator-stimulated phosphoprotein (VASP) contributes to *α*IIb/*β*_3_ activation, but its phosphorylation suppressed its activity [10, 11]. Moreover, PKA and PKG can phosphorylate IP_3_RI [12], and its phosphorylation suppresses [Ca^2+^]_i_ mobilization [13, 14].

Regarding the effects of *Cudrania tricuspidata* (*C. tricuspidata*) extracts for improving blood circulation, research has reported that the *C. tricuspidata* extract has antiplatelet effects on rat platelet aggregation [15]. Therefore, in this study, we evaluate the potential efficacy of derrone from unripe fruits of *C. tricuspidata*.

## 2. Materials and Methods

### 2.1. Reagents

Derrone was purchased from ChemFaces (Wuhan, China). Collagen was purchased from Chrono-Log Co. (Havertown, PA, USA). Fura 2-AM (2-acetoxymethyl) and Alexa Fluor 488-conjugated fibrinogen were obtained from Invitrogen (Eugene, OR, USA). Serotonin ELISA kit was purchased from Labor Diagnostika Nord GmbH and Co. (Nordhorn, Germany). Bicinchoninic acid protein assay kit was purchased from Pierce Biotechnology (IL, USA). Cayman chemical (Ann Arbor, MI, USA) offered thromboxane B_2_ assay kit, cAMP, cGMP enzyme immunoassay kit, and U46619. Cell Signaling (Beverly, MA, USA) supplied all antibodies. The adhesion kit (fibronectin coated) was obtained from Cell Biolabs (San Diego, CA, USA).

### 2.2. Human Platelets Suspension

Korean Red Cross Blood Center (Suwon, Korea) supplied human platelet-rich plasma (PRP) for research, and study protocols were approved by the Public Institutional Review Board at the National Institute for Bioethics Policy (PIRB-P01-201812-31-007, Seoul, Republic of Korea). The platelets in suspension were adjusted to 5 × 10^8^/mL concentration according to the previous research [16, 17].

### 2.3. Platelet Aggregation Analysis

For *in vitro* platelet aggregation, platelets suspension (10^8^/mL) was preincubated with derrone (15 to 60 *μ*M) at 37°C for 2 min, and then agonists were added for stimulation. Derrone was dissolved in 0.1% dimethyl sulfoxide. Platelet aggregation was measured for 5 minutes. The change in light transmission is calculated into the aggregation rate (%). Platelet aggregation was conducted using an aggregometer under stirring condition (Chrono-Log, Havertown, PA, USA).

### 2.4. Cytotoxicity Analysis

Cytotoxicity of derrone was conducted through lactate dehydrogenase leakage assay. Human platelets (10^8^/mL) was incubated with derrone (15 to 60 *μ*M) for 1 hour and centrifuged at 12,000 g. The supernatant was used to detect the lactate dehydrogenase using ELISA reader (TECAN, Salzburg, Austria).

### 2.5. Intracellular Calcium Level

The Fura 2-AM loaded platelet suspension was preincubated with derrone (15 to 60 *μ*M) at 37°C for 2 min. After incubation, we added collagen (2.5 *μ*g/mL) for calcium release from endoplasmic reticulum in platelets. The calcium mobilization was measured using a spectrofluorometer (Hitachi F-2700, Tokyo, Japan), and Grynkiewicz method was used for calculating the [Ca^2+^]_i_ concentration [18].

### 2.6. Detection of Thromboxane B_2_

Because synthesized thromboxane A_2_ (TXA_2_) is converted into thromboxane B_2_ (TXB_2_) quickly, the TXA_2_ generation was measured by detecting TXB_2_. Platelets suspension (10^8^/mL) was preincubated with derrone (15 to 60 *μ*M) at 37°C for 2 min, and then collagen (2.5 *μ*g/mL) was added for stimulation. The reaction was stopped by adding indomethacin (0.2 mM) in EDTA solution (5 mM). The TXB_2_ was detected using ELISA reader (TECAN, Salzburg, Austria).

### 2.7. Detection of Serotonin

Platelet aggregation was conducted for 7 min at 37°C with derrone (15 to 60 *μ*M), and then reaction cuvette was placed onto ice in order to terminate serotonin release for 3 min. After termination, the reaction mixture was centrifuged, and the supernatant was used. The serotonin was detected using ELISA reader (TECAN, Salzburg, Austria).

### 2.8. Immunoblotting

After platelet aggregation, platelets are dissolved using lysis buffer. The amount of dissolved protein was calculated, and proteins (15 *μ*g) were divided by 8% SDS-PAGE. After electrophoresis, proteins are transferred onto membranes and treated primary (1 : 1,000) and secondary antibodies (1 : 10,000). Western blotting analysis was conducted by Quantity One program (BioRad, Hercules, CA, USA).

### 2.9. Fibrinogen Binding to *α*_IIb_*β*_3_

After platelet aggregation for 7 min, the reaction mixture was incubated with fibrinogen (Alexa flour 488-conjugated) for 5 mins. After incubation, 0.5% paraformaldehyde in PBS was added to fix the binding between platelet integrin and fibrinogen marker. All procedures of fibrinogen binding assay were conducted in the dark condition. The flow cytometry measures the binding (BD Biosciences, San Jose, CA, USA).

### 2.10. Fibronectin Adhesion

Human platelet suspension (10^8^/mL) was placed in fibronectin coated wells (bovine serum albumin coated well is used as a negative control) and preincubated with derrone (15 to 60 *μ*M) and collagen (2.5 *μ*g/mL) for 1 h at 37°C. After incubation, wells were washed using PBS buffer and added cell stain solution for 10 min. After that, extraction solution was added, and each extraction was measured by ELISA reader (TECAN, Salzburg, Austria).

### 2.11. Fibrin Clot Retraction

Human platelet-rich plasma (300 *μ*L) was incubated with derrone (15 to 60 *μ*M) for 30 min at 37°C, and thrombin (0.05 U/mL) triggers the clot retraction. After reacting for 15 min, pictures of fibrin clot were taken using a digital camera. ImageJ (v1. 46) was used to convert to clot area (National Institutes of Health, USA).

### 2.12. Statistical Analyses

All data are presented as the mean ± standard deviation with various numbers of observations. To determine major differences among groups, analysis of variance was performed, followed by the Tukey–Kramer method. SPSS 21.0.0.0 software (SPSS, Chicago, IL, USA) was used for statistical analysis, and *p* < 0.05 was considered statistically significant.

## 3. Results

### 3.1. Inhibitory Action of Derrone on Platelet Aggregation and Cytotoxicity

**Figure 1:**
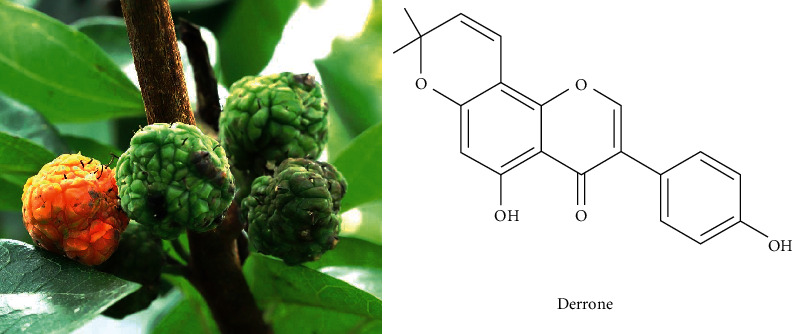
Unripe fruits of *Cudrania tricuspidata* and chemical structure of derrone.

**Figure 2:**
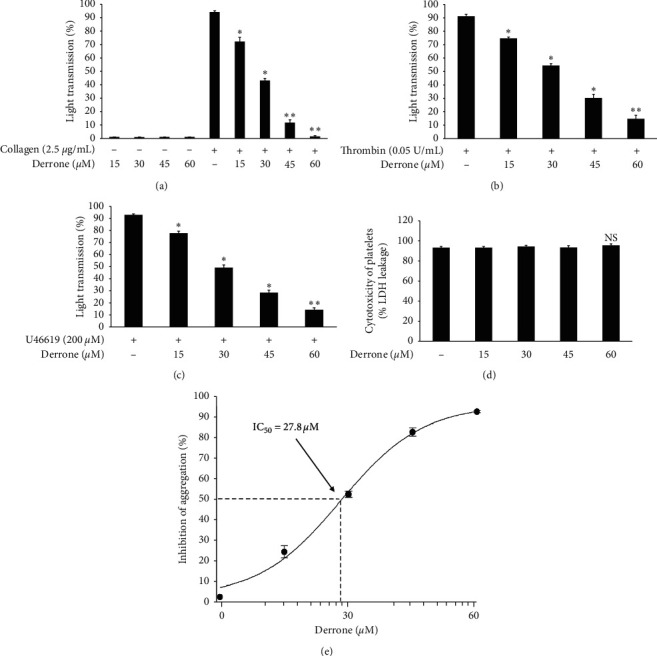
Inhibitory action of derrone on platelet aggregation, cytotoxicity, and half maximal inhibitory concentration. (a) Inhibitory action of derrone on collagen-induced human platelet aggregation. (b) Inhibitory action of derrone on thrombin-induced human platelet aggregation. (c) Inhibitory action of derrone on U46619-induced human platelet aggregation. (d) Action of derrone on cytotoxicity. (e) Half maximal inhibitory concentration (IC_50_) value of derrone in collagen-induced human platelet aggregation. Platelet aggregation and cytotoxicity were carried out as described in the Materials and Methods section. Data are expressed as mean ± standard deviation (*n* = 4).  ^*∗*^*p* < 0.05 and  ^*∗∗*^*p* < 0.01 versus each agonist-stimulated human platelets. NS, not significant.

To evaluate the antiplatelet effects of derrone, Figure 1 we used agonists: U46619, collagen, and thrombin. Collagen (2.5 *μ*g/mL), thrombin (0.05 U/mL), and U46619 (200 nM) were used for the full aggregation of human platelets (Figures 2(a)–2(c)). However, collagen-induced platelets treated with derrone (15, 30, 45, and 60 *μ*M) were the most strongly suppressed (23.3%, 54.1%, 87.5%, and 98.4%) (Figure 2(a)) without cytotoxicity (Figure 2(d)), and the half maximal inhibitory concentration (IC_50_) was 27.8 *μ*M (Figure 2(e)). DMSO 0.1% seemed to have no effect on platelet aggregation [19].

### 3.2. Inhibitory Action of Derrone on Intracellular Calcium Concentration, IP_3_RI Phosphorylation, Serotonin Secretion, and ERK Dephosphorylation

**Figure 3:**
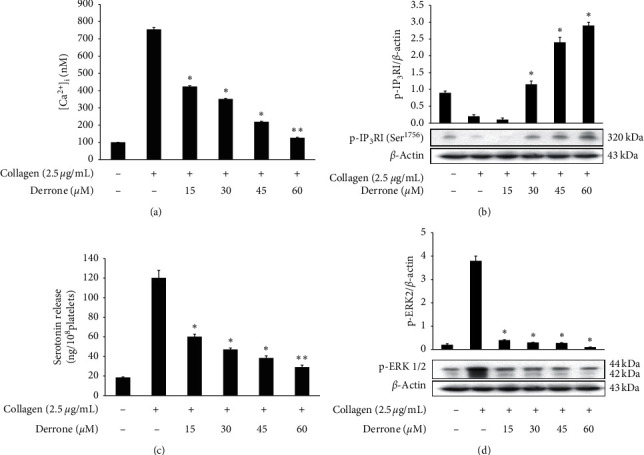
Inhibitory action of derrone on [Ca^2+^]_i_ mobilization, IP_3_RI phosphorylation, serotonin release, and ERK phosphorylation. (a) Inhibitory action of derrone on collagen-induced [Ca^2+^]_i_ mobilization (b) inhibitory action of derrone on collagen-induced IP_3_RI (Ser^1756^) phosphorylation. (c) Inhibitory action of derrone on collagen-induced serotonin release. (d) Inhibitory action of derrone on collagen-induced ERK 1/2 phosphorylation. Measurement of [Ca^2+^]_i_ mobilization and serotonin release and western blotting were performed as described in the Materials and Methods section. Data are expressed as mean ± standard deviation (*n* = 4).  ^*∗*^*p* < 0.05 and  ^*∗∗*^*p* < 0.01 versus the collagen-stimulated human platelets.

As shown in Figure 3(a), the intracellular calcium level ([Ca^2+^]_*i*_) was elevated from 100.5 ± 0.4 nM to 755.5 ± 10.2 nM by collagen (2.5 *μ*g/mL). However, derrone (15 to 60 *μ*M) reduced the collagen-increased [Ca^2+^]_*i*_ levels (Figure 3(a)). Next, we investigated the phosphorylation of calcium-mobilization signaling molecules. As shown in Figure 3(b), derrone (15 to 60 *μ*M) increased inositol 1, 4, 5-triphosphate receptor type I (IP_3_RI) phosphorylation. This shows that derrone decreased [Ca^2+^]_*i*_ levels through phosphorylation of IP_3_RI. In addition, we explored whether derrone is involved in the inhibition of dense granule secretion. Serotonin is not synthesized in megakaryocytes or platelets, but is taken up from plasma and stored in dense granules in platelets. Upon stimulation by agonists, high [Ca^2+^]_*i*_ levels facilitate the myosin light chain and pleckstrin phosphorylation to trigger *δ*-granule and *α*-granule release, and the serotonin released by exocytosis acts as a platelet agonist on the 5-hydroxytryptamine 2 (5-HT_2_) receptor on platelets [20]. We evaluated *δ*-granule release, and, as shown in Figure 3(c), derrone (15 to 60 *μ*M) dose-dependently inhibited collagen-stimulated serotonin secretion. It is known that ERK phosphorylation is involved in influx from extracellular Ca^2+^ [21]; therefore, we tested whether derrone is involved in ERK phosphorylation. As shown in Figure 3(d), collagen potently phosphorylated ERK2 (42 kDa) as compared to unstimulated platelets. However, derrone (15–60 *μ*M) inhibited collagen-induced phosphorylation of ERK2 (42 kDa).

### 3.3. Inhibitory Action of TXB_2_, cPLA_2_, and p38^MAPK^-Dephosphorylation

**Figure 4:**
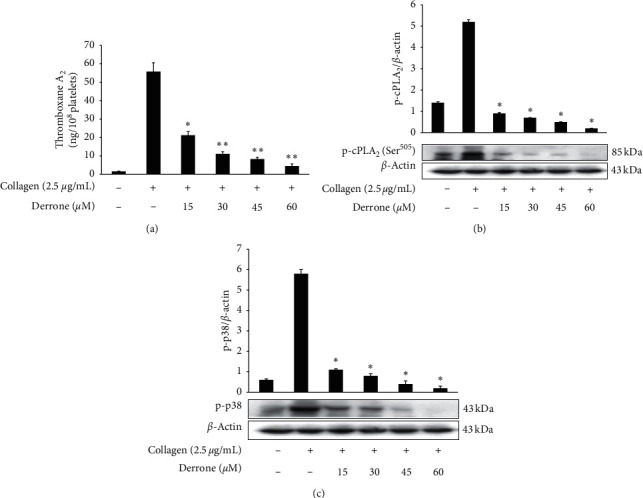
Inhibitory action of derrone on TXA_2_ generation and cPLA_2_ and p38^MAPK^ phosphorylation. (a) Inhibitory action of derrone on collagen-induced TXA2 generation. (b) Inhibitory action of derrone on collagen-induced cPLA_2_ (Ser^505^) phosphorylation. (c) Inhibitory action of derrone on collagen-induced p38^MAPK^ phosphorylation. Measurement of TXA_2_ generation and western blotting was performed as described in the Materials and Methods section. Data are expressed as mean ± standard deviation (*n* = 4).  ^*∗*^*p* < 0.05 and  ^*∗∗*^*p* < 0.01 versus the collagen-stimulated human platelets.

As shown in Figure 4(a), collagen (2.5 *μ*g/mL) increased the TXA_2_ generation to 55.8 ± 4.8 ng/10^8^ platelets from 1.5 ± 0.2 ng/10^8^ platelets. However, derrone inhibited TXA_2_ production (Figure 4(a)). To identify the inhibitory action of derrone on TXA_2_ generation, associated signaling molecules, cPLA_2,_ and mitogen-activated protein kinase p38 (p38^MAPK^), were investigated. The cPLA_2_ has been reported to hydrolyze arachidonic acid from membrane of platelets, and p38^MAPK^ activates cPLA_2_ to act as an enzyme. As shown in Figures 4(b) and 4(c), derrone suppressed cPLA_2_ and p38^MAPK^ phosphorylation in a dose-dependent manner.

### 3.4. Inhibitory Action of Derrone on Fibrinogen Binding and Fibronectin Adhesion

**Figure 5:**
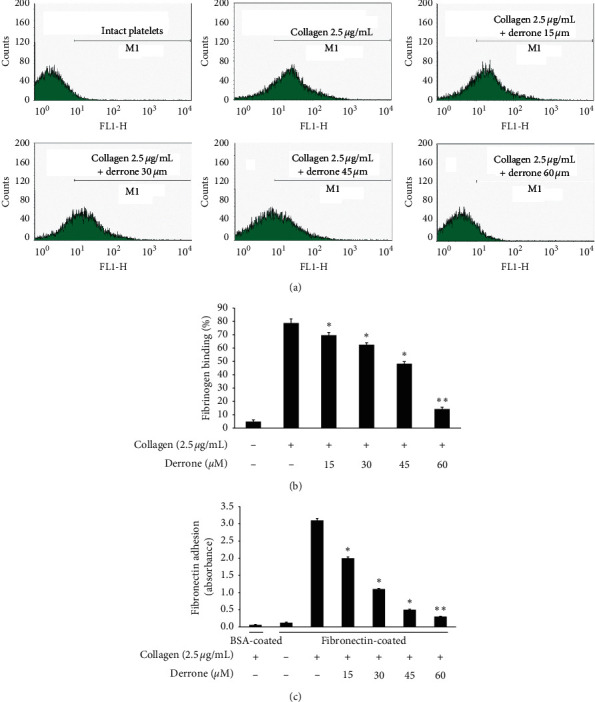
Inhibitory action of derrone on fibrinogen binding to *α*IIb/*β*3 and fibronectin adhesion. (a) Flow cytometry histograms for fibrinogen binding. (a) Intact platelets (base); (b) collagen (2.5 *μ*g/mL); (c) collagen (2.5 *µ*g/mL) + derrone (15 *μ*M); (d) collagen (2.5 *μ*g/mL) + derrone (30 *μ*M); (e) collagen (2.5 *μ*g/mL) + derrone (45 *μ*M); (f) collagen (2.5 *μ*g/mL) + derrone (60 *μ*M). (b) Inhibitory action of derrone on collagen-induced fibrinogen binding (%). (c) Effects of derrone on collagen-induced fibronectin adhesion. Measurement of fibrinogen binding and fibronectin adhesion was carried out as described in the Materials and Methods section. Data are expressed as mean ± standard deviation (*n* = 4).  ^*∗*^*p* < 0.05 and  ^*∗∗*^*p* < 0.01 versus the collagen-stimulated human platelets.

Collagen elevated *α*IIb/*β*3 affinity, the binding of fibrinogen to *α*IIb/*β*3 is increased (Figures 5(a) and 5(b)), and the binding rate is 78.8 ± 3.1%. However, derrone attenuated fibrinogen interaction with *α*IIb/*β*3 significantly (Figures 5(a) and 5(b)). *α*IIb/*β*3 can bind to fibronectin, which is essential for platelet adhesion and spreading. Thus, we examined whether derrone-treated platelets can adhere to fibronectin-coated well. As shown in Figure 5(c), derrone suppressed fibronectin adhesion.

### 3.5. Inhibitory Action of Derrone on VASP and Cyclic Nucleotides

**Figure 6:**
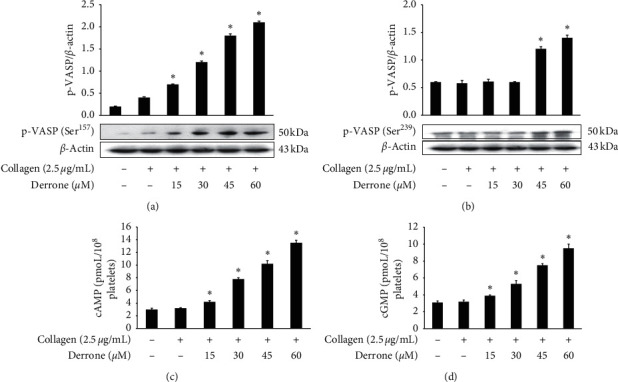
Regulation of derrone on VASP and cyclic nucleotides. (a) Regulation of derrone on collagen-induced VASP (Ser^157^) phosphorylation. (b) Regulation of derrone on collagen-induced VASP (Ser^239^) phosphorylation. (c) Regulation of derrone on collagen-induced cAMP production. (d) Regulation of derrone on collagen-induced cGMP production. Measurement of cyclic nucleotides levels and western blotting was performed as described in the Materials and Methods section. Data are expressed as mean ± standard deviation (*n* = 4).  ^*∗*^*p* < 0.05 and  ^*∗∗*^*p* < 0.01 versus the collagen-stimulated human platelets.

Vasodilator-stimulated phosphoprotein (VASP) regulates actin, but its phosphorylation by kinases inhibits *α*IIb/*β*3 affinity [10, 11]. As derrone inhibited collagen-induced *α*IIb/*β*_3_ affinity (Figures 5(a) and 5(c)), we investigated the effect of derrone on VASP phosphorylation. Derrone significantly upregulated VASP phosphorylation at Ser^157^ and VASP at Ser^239^ (Figures 6(a) and 6(b)). Next, we investigated whether derrone regulates cAMP and cGMP production in human platelets. As shown in Figures 6(c) and 6(d), derrone significantly increased cAMP and cGMP concentration.

### 3.6. Inhibitory Action of Derrone on Thrombin-Induced Clot Retraction

**Figure 7:**
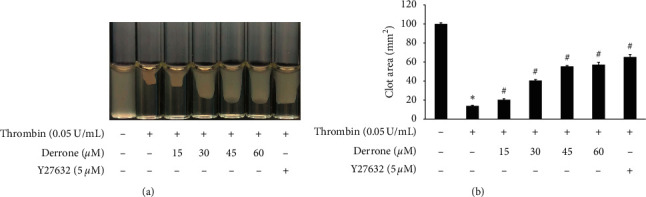
Inhibitory action of derrone on fibrin clot retraction. (a) Photographs of fibrin clot. (b) Inhibitory action of derrone on thrombin-retracted fibrin clot (%). Quantification of fibrin clot retraction was performed as described in the Materials and Methods section. Data are expressed as mean ± standard deviation (*n* = 4).  ^*∗*^*p* < 0.05 versus the unstimulated human PRP and  ^#^*p* < 0.05 versus the thrombin-stimulated human PRP.

Because derrone inhibited *α*IIb/*β*3 in the previous experiment, we examined whether derrone affects fibrin clot retraction using PRP. As shown in Figure 7(a), thrombin stimulated fibrin clot, and the clot was contracted over time. The inhibition rate was 86.1%. However, derrone (15 to 60 *μ*M) effectively delayed the thrombin-stimulated fibrin clot, with inhibitory degrees of 79.8%, 59.4%, 44.5%, 42.8%, and 34.7%, respectively (Figure 7(b)).

## 4. Discussion

*C. tricuspidata* is a perennial plant of the Moraceae family, and its roots, leaves, bark, stems, and fruits contain various physiological substances. Among the phytochemicals, xanthones and flavonoids are the major constituents of *C. tricuspidata* and have antiobesity, antidiabetic, and antitumor effects [22]. A study involving platelets, cudratricusxanthone A from *C. tricuspidata* extract has antiplatelet activity in thrombin-induced mouse platelets and anticoagulation activity [23]. Because the *C. tricuspidata* extract showed antiplatelet effects, we recently searched for a new candidate and confirmed that derrone has antiplatelet effects.

We examined whether derrone affects collagen-stimulated platelet activities and associated signaling molecules. Derrone suppressed [Ca^2+^]_i_ levels (Figure 3(a)) through phosphorylation of IP_3_RI. In addition, derrone inhibited serotonin release (Figure 3(c)), and ERK2 phosphorylation affected calcium influx (Figure 3(d)). Next, we confirmed that derrone suppressed TXA_2_ generation (Figure 4(a)). TXA_2_ is released from platelets and acts a strong agonist that affects platelet-mediated hemostasis and thrombosis. Signaling molecules involved in the production of TXA_2_ include cPLA_2_ and p38^MAPK^. p38^MAPK^ phosphorylates cPLA_2_ for full catalytic activity, and cPLA_2_ binds with Ca^2+^ [24, 25]. Derrone inhibited the phosphorylation of p38^MAPK^ and cPLA_2_ (Figures 4(b) and 4(c)), which influenced TXA_2_ generation (Figure 4(a)).

On the platelet surface, *α*IIb/*β*_3_ is the most abundant receptor and is an important binding and adhesion molecule for fibrin-platelet mesh construction and platelet-monocyte interaction. The activation of *α*IIb/*β*_3_ on the platelet membrane leads to its rapid conformational change, allowing binding to adhesive molecules. Derrone suppressed *α*IIb/*β*3 affinity, leading to binding and adhesion (Figures 5(a)–5(c)) by upregulating the phosphorylation of VASP (Figures 6(a) and 6(b)) and elevating cAMP and cGMP levels (Figures 6(c) and 6(d)). Moreover, inside-out signaling pathway activates *α*IIb/*β*3 and leads to clot retraction. In our experiment, derrone suppressed *α*IIb/*β*3 affinity, and clot retraction (Figure 7(a)), through VASP phosphorylation (Ser^157^ and Ser^239^). These data indicate that regulation of signaling molecules such as IP_3_RI (Ser^1756^) and VASP (Ser^157^, Ser^239^) influences the clot retraction delay.

According to the results of studies on the antiplatelet effects of *C. tricuspidata,* administration of extract of *C. tricuspidate* (50 and 100 mg/kg) reduced hyperaggregated platelet aggregation without any hepatotoxicity in high-fat diet- (HFD-) fed rats. Moreover, a decrease in thromboxane *A*_2_ production was observed *in vivo* [15]. In addition, we confirmed the antiplatelet effects of cudraxanthone *L*, euchrestaflavanone A, and cudraxanthone B [26–28]. Their inhibitory mechanism was similar to that of derrone, but cudraxanthone *L* and euchrestaflavanone A increased only the cAMP levels.

Our study had some limitations, in that it was conducted *in vitro* and did not confirm the antiplatelet effect *in vivo*. Although the experiment was conducted using a low concentration (15–45 *μ*M) of derrone *in vitro*, it is difficult to reach this concentration in blood by ingestion. Moreover, since our study is not an *in vivo* study using the human body, it is difficult to prove its effect in the human body; thus, we cannot prove that these effects would be the same in people with high and low platelet counts or cardiovascular disease. However, based on the *in vitro* effect, we suggest that derrone has the potential to inhibit thrombosis-mediated CVDs.

In conclusion, we confirmed that derrone suppressed collagen-stimulated platelet aggregation through downregulation of intracellular calcium concentration, *α*IIb/*β*_3_ affinity, and clot retraction, which were achieved by the regulation of IP_3_RI (Ser^1756^), VASP (Ser^157^ and Ser^239^), cPLA_2_ (Ser^505^), and p38^MAPK^. In addition, derrone increases cAMP and cGMP levels in human platelets. These two cyclic nucleotides are key mediators of the antiplatelet effects. Thus, we confirmed that derrone could be a potential phytochemical for the prevention of platelet-mediated illnesses.

## Data Availability

The data used to support the findings of this study are included within the article.
